# Design of Polymeric Nanocapsules for Intranasal Vaccination against Mycobacterium Tuberculosis: Influence of the Polymeric Shell and Antigen Positioning

**DOI:** 10.3390/pharmaceutics12060489

**Published:** 2020-05-28

**Authors:** Lara Diego-González, José Crecente-Campo, Matthew John Paul, Mahavir Singh, Rajko Reljic, María José Alonso, África González-Fernández, Rosana Simón-Vázquez

**Affiliations:** 1 Inmunología, Centro de Investigaciones Biomédicas, CINBIO, Universidade de Vigo, Campus Universitario Lagoas Marcosende, 36310 Vigo, Spain; ldiego@uvigo.es (L.D.-G.); africa@uvigo.es (Á.G.-F.); 2 Instituto de Investigación Sanitaria Galicia Sur (IIS-GS), SERGAS-UVIGO, Estrada de Clara Campoamor, 341, 36312 Vigo, PO, Spain; 3 Department of Pharmacology, Pharmacy and Pharmaceutical Technology, School of Pharmacy, Campus Vida, Center for Research in Molecular Medicine and Chronic Diseases (CIMUS), IDIS Research Institute, Universidade de Santiago de Compostela, 15782 Santiago de Compostela, Spain; mariaj.alonso@usc.es; 4 Institute for Infection and Immunity, St George’s Medical School, London SW17 0RE, UK; mpaul@sgul.ac.uk (M.J.P.); rreljic@sgul.ac.uk (R.R.); 5 Lionex GmbH, 38126 Braunschweig, Germany; info@lionex.de

**Keywords:** 6 kDa early secretory antigenic target (ESAT-6), 10 kDa culture filtrate protein (CFP-10), vaccination, Imiquimod, Toll-like receptor-7 (TLR-7), antibodies, cytokines, complement system, reactive oxygen species (ROS)

## Abstract

Tuberculosis (TB) is the leading cause of death from a single infectious microorganism and Bacillus Calmette Guerin (BCG), the only authorized vaccine, does not confer protection against pulmonary TB. Based on the hypothesis that mucosal protection could help to prevent the infection at the site of entrance, the objective of this work was to develop an intranasal vaccine against *Mycobacterium tuberculosis* (Mtb), the microorganism that causes TB. Our approach consisted of the use of polymeric nanocapsules (NCs) with an oily core and a polymer shell made of chitosan (CS) or inulin/polyarginine (INU/pArg). The immunostimulant Imiquimod, a Toll-like receptor-7 (TLR-7) agonist, was encapsulated in the oily core and a fusion protein, formed by two antigens of Mtb, was absorbed either onto the NC surface (CS:Ag and INU:pArg:Ag) or between two polymer layers (INU:Ag:pArg) in order to assess the influence of the antigen positioning on the immune response. Although CS NCs were more immunostimulant than the INU/pArg NCs in vitro, the in vivo experiments showed that INU:pArg:Ag NCs were the only prototype inducing an adequate immunoglobulin A (IgA) response. Moreover, a previous immunization with BCG increased the immune response for CS NCs but, conversely, decreased for INU/pArg NCs. Further optimization of the antigen and the vaccination regime could provide an efficacious vaccine, using the INU:pArg:Ag NC prototype as nanocarrier.

## 1. Introduction

The use of vaccines to prevent severe infectious diseases has been a historical landmark in medicine. However, some pathogens remain elusive to the development of an efficient vaccine against them, such as the case of tuberculosis (TB), the most deadly infectious disease in the world, caused by *Mycobacterium tuberculosis* (Mtb) [1]. The BCG vaccine, containing the Bacillus Calmette Guerin, which is the only one licensed to date for TB, protects against non-pulmonary TB in infants, however, it is unreliable in protecting against pulmonary TB, which accounts for most of the disease burden worldwide [2]. Approved vaccines based on live-attenuated or inactivated pathogens provide a good immunogenicity in general, but the risk associated to their administration is relevant. For that reason, subunit vaccines are preferred due to their inherent safety, although they show limited immunogenicity [3]. Moreover, the adjuvants available on the market, mainly based on aluminum salts, have failed to induce an efficient immune response against some antigens, due to a biased or a suppressive immune response, among other factors [4]. 

For these reasons, new strategies to stimulate the immune system towards better protective responses are strongly needed. In this sense, nanotechnology offers the possibility to develop more powerful vaccines. This is because the association of antigens to nanocarriers enables their protection against degradation and improves their presentation to the immune system [5,6]. 

Polymer- and lipid-based nanocarriers are among the most widely used nanocarriers for vaccine development due to, among other properties, their biocompatibility and biodegradability, the capacity of some polymers and lipids to interact with pattern-recognition receptors (PRRs) or cell membranes, and their capacity to enhance both humoral and cellular immune responses [5,7,8,9,10]. In particular, polymeric nanocapsules (NCs) have been shown to be promising carriers for the delivery of a variety of antigens against different pathogens [11,12,13].

In most vaccines, a balanced type 1 T helper / type 2 T helper (Th1/Th2) response is desired to trigger a wide-ranging immune response and, consequently, protective efficacy [8,14]. The immunogenicity of the nanosystems can be further enhanced by including small immunostimulant molecules in the particle structure [4]. In this sense, Imiquimod (IMQ) has been described as a good modulator of the innate immunity and activator of the Th1 immune response via binding to the Toll-like receptor-7 (TLR-7) on antigen presenting cells (APCs). Previous work from our laboratory has shown that encapsulation of IMQ in chitosan (CS) NCs induced protective antibody levels against the recombinant hepatitis B surface antigen (HB) in mice immunized by the intranasal (i.n.) route [8]. Interestingly, the i.n. route could also induce additional protection at the mucosal level, with the production of immunoglobulin isotype A (IgA) antibodies and activation of local immune cells [15]. Prompt, appropriate mucosal immune responses could be very helpful to neutralize pathogens at their main route of entrance, such as in the case of Mtb, avoiding the development of the infection altogether.

Having this background in mind, the goal of this work was to develop polymeric NCs containing the immunostimulant IMQ and a fusion protein antigen of the 6 kilodaltons (kDa) early secretory antigenic target (ESAT-6) and the 10 kDa Culture Filtrate Protein (CFP-10) against Mtb to be administered intranasally. To study the effect of the polymeric shell and antigen distribution on the immunogenicity of an i.n. vaccine, we selected two different NCs. CS and inulin/polyarginine (INU/pArg) were selected as polymeric shell for the first and second NC prototypes, respectively. Moreover, in the INU/pArg NCs, the antigen was added on the surface of the pArg polymer shell or between the two polymer layers to assess the influence of the antigen positioning on the immune response. In fact, the entrapment of the antigen in a bilayer disposition of polymeric NCs has recently been shown to offer adequate protection and an enhanced immune response towards the associated antigen [11].

The biocompatibility and the immunostimulant properties of the NCs were tested in vitro with different cell lines and human peripheral blood mononuclear cells (PBMCs). Finally, the immunogenicity of the vaccine prototypes by the i.n. route was tested either in naïve mice or in mice previously immunized (subcutaneously, s.c.) with the BCG vaccine, to test the synergy between the conventional BCG vaccine and the polymeric NC vaccines. 

## 2. Materials and Methods 

### 2.1. Materials for the Synthesis of the Nanocapsules (NCs)

Ultrapure CS hydrochloride salt (CS.HCl) (Molecular weight (Mw) 42.7 kilodaltons (kDa), deacetylation degree of 88%) was purchased from Heppe Medical Chitosan GmbH (Saale, Germany). Inutec^®^SL1 (25% modified inulin (INU) suspension in glycerol) was a kind gift from CreaChem (Tienen, Belgium).

PArg (Mw 24 kDa) was purchased from PTS (Valencia, Spain) and Imiquimod was provided by Ferrer HealthTech (Barcelona, Spain). 

Sodium glycocholate and sodium cholate were purchased from Dextra (Reading, UK).

Miglyol^®^ 812 was donated by Cremer Oleo GmbH & Co (Hamburg, Germany) and linoleic acid was provided by Acros Organics, Thermo Fisher (Waltham, MA, USA). 

The 1,2-distearoyl-sn-glycero-3-phosphoethanolamine-N-[methoxy (polyethylene glycol) 1000] (18:O PE-PEG1000) was purchased from Avanti Polar Lipids Inc. (Alabaster, AL, USA). 

Sucrose and trehalose were purchased from Acofarma (Madrid, Spain).

ESAT-6/CFP-10 fusion protein (ECH) was provided by Lionex Gmbh (Braunschweig, Germany, www.lionex.de). 

All solvents employed were of analytical grade and supplied by Merck Organic solvents (Madrid, Spain). Endotoxin-free water was collected in a Millipore Ultrapure Water System equipped with a filter unit for endotoxin removal.

### 2.2. Nanocapsules’ Synthesis and Characterization

The NCs were synthetized by the solvent-displacement method, as described previously [16,17]. 

For CS NCs, the organic phase was prepared with 125 µL of a mixture of linoleic acid an Miglyol^®^ 812 (9.5:3, *v/v* ratio) and 1 mg of IMQ, 20 mg of the PEGylated phosphoethanolamine 18:0 PE-PEG1000 and 25 µL of an aqueous solution of 200 mg/mL sodium cholate in 5 mL of ethanol. The aqueous phase contained 5 mg of CS.HCl salt dissolved in 10 mL of water. After pouring the organic phase over the aqueous phases, stirring the suspension and evaporating the organic solvent in a rotavapor (Büchi, Switzerland), the suspension was taken to a final volume of 5 mL with ultrapure water. 

For the INU/pArg NCs, the organic phase was formed by 34.5 µL of a mixture of linoleic acid and Miglyol^®^ 812 (6.5:1, *v/v* ratio) and 1 mg of IMQ, 5 mL of ethanol, and 25 µL of an aqueous solution of sodium glycocholate at 200 mg/mL. The aqueous phase was prepared by dissolving 15 mg of Inutec SL1 in 10 mL of water. The NCs were prepared as described above for the CS NCs. Afterwards, 0.5 mL of a pArg aqueous solution at 10 mg/mL was added to the INU NCs, before or after the addition of the antigen, to form the INU:pArg:Ag and the INU:Ag:pArg NCs, respectively. The formulation was shaken for 1 h at 300 rpm in a thermoblock (Eppendorf Thermomixer R) to allow the adsorption of the positively charged pArg over the negatively charged INU core by electrostatic interactions.

Lyophilization studies of the final NC prototypes were performed in the presence of 5 and 10% sucrose or trehalose, as cryoprotectant.

Particle size and polydispersity index (PdI) of the formulations in ultrapure water were characterized by photon correlation spectroscopy in a Zetasizer Nano-S (Malvern Instruments; Malvern, UK) at 25 °C. Zeta potential (Z-potential) was also measured in the same equipment by laser Doppler anemometry (LDA) using an appropriate cell and 1 mM KCl as solvent. 

The IMQ encapsulated in the oily core was determined after ultracentrifugation (30,000 rpm, 1 h, 15 °C) of the NCs to separate the undernatant, containing the free IMQ (Cf), from the cream, with the encapsulated IMQ (Ce). Besides, total IMQ concentration (Ct) was also measured in initial NCs. All samples were diluted 1:10 with MeOH to break the NCs and solubilize the IMQ. Subsequently, the samples were analyzed by HPLC, as described in [8]. A calibration curve was also made up with standard solutions of IMQ to determine the concentration in each sample. The encapsulation efficiency (%EE) and the drug loading (%DL) were quantified directly according to the formula:

%EE = (Ce/Ct) × 100, %DL = [IMQ (mg)/NCs (mg)] × 100(1)

### 2.3. Adsorption of the Antigen onto the Nanocapsules and Quantification of the Adsorbed Protein

For the development of the model vaccine, a preliminary screening was performed, adding the ECH fusion protein to the NCs at different ratios (polymer:antigen, *w/w*), to find the maximum antigen loading which did not compromise the NCs colloidal stability. The antigen was added to the IMQ-loaded NCs and the mixture was incubated at room temperature with constant orbital shaking (300 rpm) for one hour in a thermoshaker (Eppendorf Thermomixer R) to allow the protein adsorption to the polymer surface. The amount of antigen adsorbed on the surface of IMQ-loaded NCs (CS:Ag and INU:pArg:Ag) or entrapped between two polymer layers (INU:Ag:pArg) was determined by SDS-PAGE and Coomassie blue staining. The NCs were ultracentrifuged (30,000 rpm, 1 h, 15 °C) to separate free (ECHf) from attached protein (ECH.NCs) and an aliquot of both fractions was loaded in a 10% polyacrylamide gel. A molecular marker was also loaded in a different lane to follow the protein separation and to check the ECH protein molecular weight. After running the electrophoresis, the gel was stained and the density of the protein bands was quantified in a ChemiDoc equipment using the LabImage software (Bio-Rad Laboratories, Inc, Hercules, CA, USA). The percentage of absorbed antigen (%AA) was calculated according to the formula:

%AA = ECH.NCs/(ECH.NCs + ECHf)(2)

### 2.4. Materials for Protein, Cell, and Animal Studies

ESAT-6 and CFP-10 antigens were provided by Lionex Gmbh (Braunschweig, Germany, www.lionex.de). 

Mouse monoclonal antibody (mAb) against human complement factor C3 and the split protein C3b (C3-C3b) was from Abcam, (Cambridge, UK) and polyclonal goat anti-mouse IgG antibodies (Abs) conjugated with alkaline phosphatase (AP) were from Dako (Glostrup, Denmark).

The 5-bromo-4-chloro-3-indolyl-phosphate (BCIP)/nitro blue tetrazolium (NBT) substrate solution, 2′,7′-dichlorodihydrofluorescindiacetate (H_2_DCFDA), anti-mouse IgA, anti-mouse IgG, and o-phenylenediamine dihydrochloride (OPD) substrate were purchased from Sigma-Aldrich Co. and molecular weight marker was from Thermo Fisher Scientific, Spain. 

Cell Titer 96^®^ AQueous One Solution Cell Proliferation Assay kit, 3-(4, 5-dimethylthiazol-2-yl)-5-(3-carboxymethoxyphenyl)-2-(4-sulfophenyl)-2H-tetrazolium (MTS), and endotoxin test were from Promega Biotech Ibérica, (Madrid, Spain) and Associates of Cape Cod, Inc. (Falmouth, MA, USA), respectively.

Phorbol 12-myristate-13-acetate (PMA) was from Abcam (Cambrige, UK), lipopolysaccharide (LPS) from InvivoGen (Toulouse, France), and phytohaemagglutinin (PHA) from Sigma-Aldrich Co.

The MILLIPLEX^®^ MAP Kit used for cytokine profile characterization was provided by Millipore, Merck KGaA (Darmstadt, Germany).

### 2.5. Cells and Culture Conditions 

Five different cell lines from the American Type Culture Collection (ATCC) (Manassas, VA, USA) were used: Raw 264.7 (mouse macrophages), THP-1 (human monocytes), A549 and NCI-H460 (human lung epithelial cells), and HL-60 (human promyeloblast cells).

The cells were cultured in Roswell Park Memorial Institute medium (RPMI) supplemented with 10% (*v/v*) heated, inactivated fetal bovine serum (FBS) (PAA; Pasching, Austria), 2 mM glutamine and 100 U/mL of penicillin/streptomycin, at 37 °C in 5% CO_2_ atmosphere. Cells were diluted every two or three days, after reaching a 70–80% of confluence.

### 2.6. Evaluation of Bacterial or Endotoxin Contamination

The absence of bacterial contamination was evaluated by seeding 100 µL of the samples on Luria-Bertani (LB) agar plates under aseptic conditions. The plates were incubated at 37 °C for 24–72 h. Potential endotoxin contamination on the NC formulations was also assessed by the gel-clot assay, following the manufacturer’s instructions.

### 2.7. Toxicity Studies on Macrophages and Lung Epithelial Cells

Cell viability tests were performed in two macrophage (RAW 264.7 and PMA-differentiated THP-1) and two lung epithelial (A549 and NCI-H460) cell lines. 

The colorimetric MTS test was used to quantify the changes on cell viability after 24 h of incubation with different concentrations of the IMQ-loaded NCs.

The cells were seeded on 96-well plates at a density of 1.5 × 10^4^ cells/well for THP-1 and RAW264.7, and 6 × 10^3^ and 7 × 10^3^ cells/well for A549 and NCI-H460, respectively. THP-1 cells were incubated with phorbol 12-myristate-13-acetate (PMA) at 10 ng/mL for 24 h to induce their differentiation into macrophages. The medium with PMA was replaced with fresh medium before adding the NCs. The maximum concentration tested for CS and INU/pArg NCs was 1440 and 545 µg/mL, respectively. Several dilutions (1:2) of each NC prototype were also tested in order to determine the half-maximun inhibitory concentration (IC50). Culture medium alone and with NCs was used for background subtraction and to discard any interference of the NCs in the absorbance. The percentage of cell viability was calculated using the following Equation:

%Cell viability = [(Abs Cells&NCs − Abs NCs)/(Abs Cell − Abs Medium)] × 100(3)

### 2.8. Production of Reactive Oxygen Species (ROS)

For the characterization of ROS release, the promyeloblast cell line HL-60 was used. The cells were seeded at a density of 1 × 10^5^ cells/well in a 96-well plate and incubated with the NCs during 1, 3, 6, and 14 h. The NCs were tested at three different concentrations (50, 100, and 200 µg/mL for CS NCs and 10, 50, and 100 µg/mL for INU:pArg NCs).

PMA at 20 µM was added to the cells as positive control and cells incubated only with culture medium were used as negative control. After the corresponding incubation time, H_2_DCFDA at 2.5 µM was added to the cells and incubated for 30 min at 37 °C. After diffusion into the cell and oxidation to 2′,7′-dichlorofluorescein (DCF) by ROS in a concentration-dependent rate, the cells were washed and the fluorescence produced by DCF was measured in a Beckman Coulter FC500 flow cytometer (Beckman Coulter, IN, USA). Cells were also labelled with propidium iodide (PI) to discriminate live and dead populations. 

### 2.9. Analysis of Complement Activation

Complement activation induced by the IMQ-loaded NCs in a pool of plasma from healthy donors was assessed by measuring the degradation of the C3 factor by Western blot. 

Equal volumes of plasma, PBS, and samples were mixed and incubated at 37 °C for 1 h. Zymosan at a final concentration of 1 mg/mL and PBS were used as positive and negative controls, respectively. The NCs were tested at three different concentrations (50, 100, and 200 µg/mL for CS NCs and 10, 50, and 100 µg/mL for INU/pArg NCs).

After incubation, supernatants were collected and 2 µL were mixed with sample buffer and loaded onto a 10% SDS-PAGE gel and transferred to a Polyvinylidene Difluoride (PVDF) membrane using the Transblot Semidry Transfer Equipment (Bio-Rad Laboratories, Inc). 

The PVDF membranes were blocked with 5% nonfat milk in Tris-buffered saline in Tween 20 (TBS-T), and incubated sequentially with mouse monoclonal antibody (mAb) against human C3-C3b followed by polyclonal goat anti-mouse IgG antibodies (Abs) conjugated with alkaline phosphatase (AP), both diluted 1:2000 in 5% nonfat milk in TBS-T. Each incubation was carried out for 1 h at room temperature and the membranes were washed three times with TBS-T between each incubation. The bands of the intact and split C3 protein were visualized adding a BCIP/NBT substrate solution that reacted with the AP and formed a dark precipitate. The intensity of the bands was quantified on the ChemiDoc (Bio-Rad Laboratories, Inc). The ratio of split/intact protein was normalized to the negative control.

### 2.10. Cytokine Profile Evaluation

Cytokines’ release was studied in human PBMCs from three healthy donors. The PBMCs were obtained from whole blood by density gradient centrifugation. After centrifugation, cells were washed with PBS and seeded at a density of 1.75 × 10^6^ cells/mL in 96-well U-bottom plates. The cells were allowed to rest for 24 h in the incubator before adding the samples.

The NCs were added at the same final concentrations as in the previous experiment and the plate was incubated for another 24 h. As positive control, lipopolysaccharide (LPS) at 1 µg/mL with phytohaemagglutinin (PHA) at 10 µg/mL was used and cells without any treatment were used as negative control.

After incubation with the NCs, the plate was centrifuged at 1200 rpm for 5 min and 100 µL of supernatant per well were recovered and stored at −80 °C until measurement. 

A MILLIPLEX^®^ MAP Kit for 11 cytokines (granulocyte monocyte-colony stimulating factor (GM-CSF), interferon (IFN)-γ, interleukin (IL)-10, IL-12, IL-17, IL-1β, IL-2, IL-4, IL-5, IL-6, and tumor necrosis factor (TNF)-α was used to measure the concentration of these cytokines in the supernatants collected from the PBMCs using a MAGPIX^®^ instrument (Millipore, Merck KGaA, Darmstadt, Germany) and following the manufacturer’s instructions. The MAGPIX^®^ software was used for the analysis of the standard curves and the samples.

Mouse IFN-γ, TNF-α, and IL-17 release by the splenocytes of immunized animals after antigen recall was monitored by enzyme-linked immunosorbent assay (ELISA) (Invitrogen 88-7314-86, 88-7324-77, 88-7371-77, ThermoFisher Scientific, Loughborough, UK).

### 2.11. Immunization Experiments 

Female C57BL/6 mice were obtained from Charles River, UK, and were between 8 and 12 weeks of age before experimental use. In the groups specified, mice were subcutaneously immunized with 5 × 10^5^ CFU BCG Pasteur (100 μL) or vehicle control. After 12 weeks, intranasal booster immunizations were given consisting of 3x IMQ-loaded polymeric NCs coated with 10 μg ECH in 50 μL per animal per dose, or vehicle control. Doses were given 2 weeks apart. These immunizations were performed under light anesthesia. Three weeks following the final immunization, animals were culled by intraperitoneal (i.p.) administration of 200 mg/kg pentobarbitone solution (Pentoject^®^, Animalcare Ltd., York UK). Bronchoalveolar fluid was collected by passing 1 mL of PBS into the lungs using a 22-gauge catheter positioned in the trachea.

### 2.12. Detection of Specific Antibodies 

Antibody levels were quantified by ELISA. Antigens [ECH, ESAT-6, and CFP-10] were coated onto a plate at 2 μg/mL overnight, followed by blocking for 2 h with PBS containing 1% bovine serum albumin (BSA). Serum samples were loaded at a 1:10 dilution in PBS with 1% BSA and 4-fold serial dilutions were made. Bronchoalveolar lavage fluid (BAL) samples were loaded undiluted and 3-fold serial dilutions were performed. All samples were incubated on the plate in triplicate for 1 h at 37 °C. Levels of IgA (BAL) or IgG (serum) were detected using peroxidase-conjugated anti-mouse IgA or anti-mouse IgG and OPD substrate. Plates were read on a Tecan Infinite F200 Pro plate-reader at 450-nm absorbance.

### 2.13. Statistical Analysis

The GraphPad Prism 8 software (GraphPad Software Inc., San Jose, CA, USA) was used for data representation and statistical analysis. Each experiment was performed at least three times (n ≥ 3) and the results of the different assays were represented as mean ± standard deviation (SD). T-student or Mann–Whitney test was performed to determine significant differences between negative control and treatments. In the graphs, results are indicated as: * P ≤ 0.05, ** P ≤ 0.01, *** P ≤ 0.001, **** P ≤ 0.0001.

## 3. Results

### 3.1. Physicochemical Characterization and Stability of the Nanocapsules

**Figure 1:**
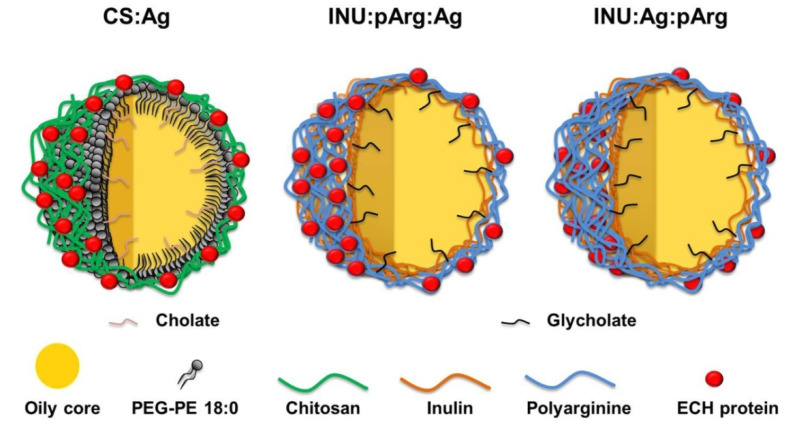
Polymeric vaccine prototypes designed for the development of an intranasal vaccine against the *Mycobacterium tuberculosis* fusion protein ESAT-6/CFP-10 (ECH protein). In the oily core, Imiquimod, a TLR-7 agonist, was added as adjuvant to increase the immunogenicity of the fusion protein. Ag: Antigen, CS: Chitosan, INU: Inulin, pArg: Polyarginine, TLR-7: Toll-like receptor-7.

During this work, three different vaccine prototypes of polymeric NCs were developed. For the first prototype, CS was used as a polymer shell because of the good biocompatibility and the proven capacity of CS-based nanocarriers to elicit mucosal and systemic immune response against a variety of antigens after i.n. administration [8,18,19,20]. The oily core consisted of a mixture of linoleic acid and Miglyol^®^ 812. PEGylated phosphoethanolamine 18:0 and sodium cholate were used as surfactant and as co-surfactant, respectively. The antigen was absorbed onto the surface by incubating the antigen solution with the preformed NCs. The adsorption was probably caused by a combination of electrostatic and hydrophobic interactions (Figure 1 and Table 1).

For the second prototype, pArg was selected as a polymer shell due to the penetrating properties of this polypeptide, which could help crossing the mucosal barrier [21]. In the pArg NCs, a first layer of hydrophobized INU and sodium glycocholate were used to increase the stability of the naoemulsion (NE), whose core was again a mixture of linoleic acid and Miglyol^®^ 812. Afterwards, antigen was adsorbed onto the pArg surface. However, the antigen could also be added at the surface of the INU before adding the pArg shell, entrapping it in a sandwich-like arrangement [11]. This engineering strategy allowed obtaining two different vaccine prototypes, one with the antigen adsorbed on the surface (INU:pArg:Ag) and another one with the antigen protected by a polymeric bilayer (INU:Ag:pArg) (Figure 1 and Table 1), which can influence the vaccine performance, the reason why we considered it interesting to be studied [11].

To enhance the immunostimulatory properties of the NCs, IMQ, a lipophilic immunostimulant, was incorporated in the oily core of the nanosystems. The IMQ-loaded polymeric NCs, both for CS and pArg prototypes, had a particle size about 150 nm and a PdI lower than 0.2 (Table 2). The surface charge was positive for CS and INU/pArg NCs and negative for INU NCs (Table 2), consistent with the positive charge of CS and pArg polymers and the negative charge of the cholate-stabilized INU NCs.

The selected antigen was a fusion protein of two Mtb antigens, ESAT-6 and CFP-10, named ECH protein, with a molecular weight of approximately 21.5 kDa and a theoretical isoelectric point (pI) of 5.06. ESAT-6 and CFP-10 are virulence-related Mtb proteins which are absent in the BCG vaccine [22].

The polymer: antigen ratio 1:0.2 (*w/w*) was selected as the best ratio for both, CS and pArg NCs, based on the SDS-PAGE and dynamic light scattering (DLS) studies (data not shown). The %AA was estimated by SDS-PAGE (Table 2) and was about 60, 55, and 30% for CS:Ag, INU:Ag:pArg, and INU:pArg:Ag NCs, which corresponded to an antigen loading of 0.4, 1.1, and 0.55%, respectively. The free antigen that was not bound to the NCs allowed an initial burst release that could boost the immune response [23]. For that reason, free antigen was not removed from the suspension and, for immunization studies, the amount of antigen injected was calculated according to the initial protein concentration.

The percentage of IMQ in the NCs was measured by HPLC (as described in Section 2.2) and similar encapsulation efficiency was found for CS and INU/pArg NCs (Table 2). The encapsulation efficiency was about 60–70% in both cases, which corresponded with a drug loading of 4.2% for CS NCs and 13% for INU/pArg NCs, respectively.

**Figure 2:**
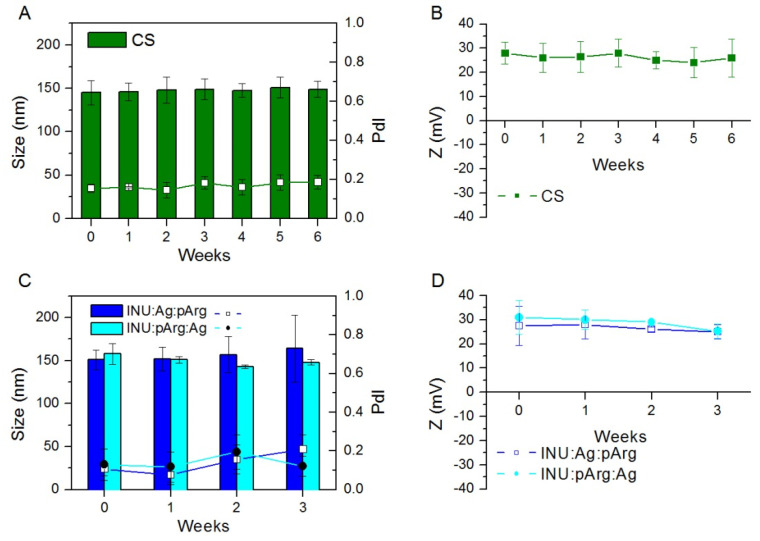
Size (bars), PdI, and Zeta-potential (lines and symbols) of Imiquimod-loaded CS (**A**,**B**), INU:Ag:pArg and INU:pArg:Ag (**C**,**D**) nanocapsules stored at 4 °C at different time points. Due to the reduced stability of the CS nanocapsules after antigen adsorption (Figure S1), the nanocapsules were tested in the absence of the antigen. Size and PdI were measured in ultrapure water and Zeta-potential in 1 mM KCl. Ag: Antigen, CS: Chitosan, INU: Inulin, pArg: Polyarginine.

The nonloaded CS NCs were stable in suspension at 4 °C for at least 6 weeks, as shown by the size and Z-potential measurements (Figure 2). However, the antigen-loaded NCs were stable only one week (Supplementary Figure S1) due to, likely, the neutralization of the CS NCs’ surface charge after addition of the ECH fusion protein (Table 2). For this reason, for all the experiments with antigen-adsorbed CS NCs, the antigen was added in situ and incubated one hour before the administration of the formulation.

The INU/pArg NCs, both with the antigen protected by the pArg shell (INU:Ag:pArg) or with the antigen on the surface (INU:pArg:Ag), were stable for three weeks at 4 °C (Figure 2). After this period, the NCs showed a tendency to undergo coalescence (data not shown). To increase their stability, the antigen-loaded CS and INU/pArg NCs were lyophilized with 5 or 10% sucrose or trehalose as cryoprotectant. Among all of them, the use of 10% sucrose proved to be effective to preserve the INU/pArg NCs during the lyophilization process and further reconstitution in water, but not for the CS:Ag NCs (Supplementary Figure S2). The lyophilized INU/pArg NCs were easily reconstituted by adding water and immediately vortexing the vial for two minutes. After reconstitution, the lyophilized NCs showed a size, a PdI, and a Zeta-potential similar to the values observed after their lyophilization. Oppositely, the size and PdI of the CS:Ag NCs increased after the lyophilization, and the use of trehalose or a lower sucrose percentage produced similar results (data not shown).

**Figure 3:**
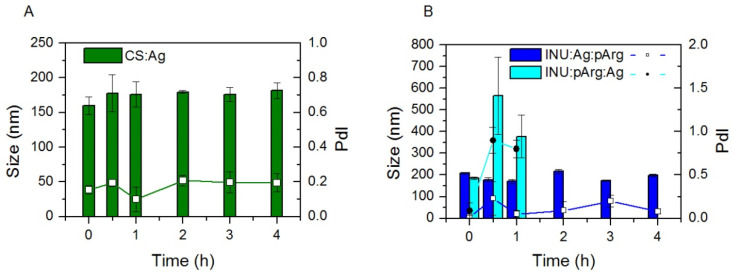
Stability of Imiquimod-loaded CS:Ag (**A**), INU:Ag:pArg, and INU:pArg:Ag (**B**) nanocapsules on RPMI culture medium supplemented with 10% FBS and incubated at 37 °C. Graphs represent the size (bars) and PdI (lines and symbols) vs. time of incubation. Ag: Antigen, CS: Chitosan, FBS: fetal bovine serum, INU: Inulin, pArg: Polyarginine.

In order to be able to draw adequate conclusions from the in vitro experiments, the stability of the NCs was tested in complete culture medium by recording size and PdI at 4 different time points (Figure 3). CS:Ag and INU:Ag:pArg NCs were stable at all the time points recorded (4 h), whereas the stability of INU:pArg:Ag NCs was limited to less than 30 min, but presumably long enough to allow particle internalization and drainage to the lymph nodes, which usually takes place in the first minutes after administration [24]. 

Hence, the protection of the antigen by the pArg shell increased the stability of the INU/pArg NCs, in comparison with the adsorption of the antigen into the surface of the NCs, in physiological medium. Conversely, CS NCs with the antigen on the surface were very stable.

### 3.2. Cytocompatibility of the Polymeric Nanocapsules in Different Cell Lines

The presence of endotoxin and bacterial contamination in the NCs was ruled out with an endotoxin test and by seeding the NCs in LB agar plates, respectively, to avoid any wrong assignment of the biological effects induced by bacterial contaminants to the NCs. The NCs were free of any source of contamination.

**Figure 4:**
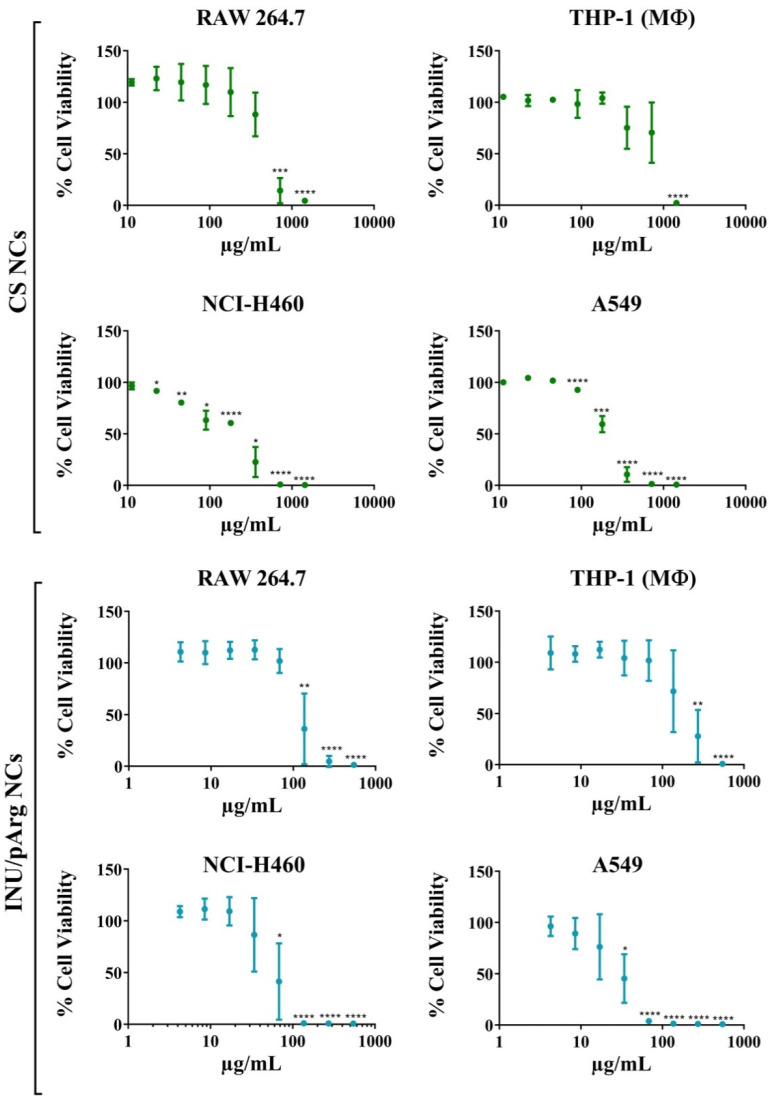
Percentage of cell viability of RAW 264.7, macrophage-differentiated THP-1, NCI-H460, and A549 incubated with different concentrations of CS and INU/pArg nanocapsules for 24 h. CS: Chitosan, INU: Inulin, pArg: Polyarginine. Statistical significance: * P ≤ 0.05, ** P ≤ 0.01, *** P ≤ 0.001, **** P ≤ 0.0001.

Since the NCs were designed for intranasal administration, two macrophage (RAW 264.7, PMA-differentiated THP-1) and two pulmonary epithelial (NCI-H460, A549) cell lines were used to test their cytocompatibility, as representative cells that can be found in the mucosal epithelium and respiratory tract. IMQ-loaded NCs were incubated at several concentrations with the cells to check the cytocompatibility of the nanosystems. At concentrations lower than 100 µg/mL, the NCs were cytocompatible with all the tested cells, except INU/pArg NCs with the pulmonary cells, which showed a lower cytocompatibility (Figure 4). Among them, the macrophage-differentiated THP-1 showed the highest IC50 and A549 the lowest one (Table 3), indicating that pulmonary epithelial cells are more sensitive to the NCs than macrophage cells. Regarding composition, CS NCs were more cytocompatible in all cell lines in comparison with INU/pArg NCs, as shown in Figure 4 and Table 3. In summary, CS and INU/pArg NCs were cytocompatible with all the cell lines tested at low-intermediate concentrations, but they were toxic at high concentrations. 

The increased toxicity of INU/pArg NCs in comparison with CS NCs must be related to their composition and, specifically, the higher IMQ content (Table 3), because both NCs were positively charged and they showed a similar size and Z-potential (Table 2). 

### 3.3. Reactive Oxygen Species’ Release in HL-60 Cells upon Exposure to the Nanocapsules

ROS release is related with cell signaling, but also with cell toxicity when their concentration is high [25]. However, at low concentration, ROS could help in antigen cross-presentation and stimulation of Th1 immune responses, which could be a useful adjuvant mechanism to induce a cellular immune response [26,27].

**Figure 5:**
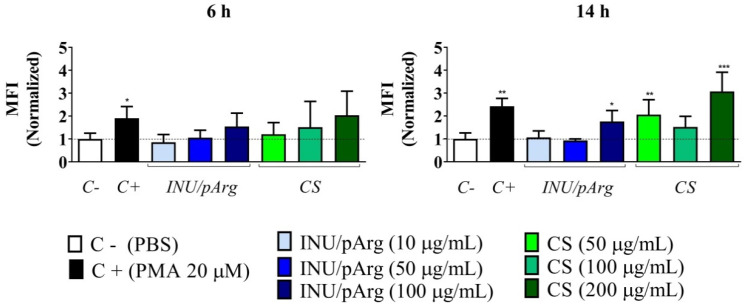
Reactive oxygen species (ROS) release in HL-60 cells incubated with CS and INU/pArg nanocapsules at three different concentrations during 6 and 14 h. CS: Chitosan, INU: Inulin and pArg: Polyarginine, PMA: Phorbol 12-myristate 13-acetate. Statistical significance: * P ≤ 0.05, ** P ≤ 0.01, *** P ≤ 0.001.

The human promyelocytic cell line, HL-60, was selected to measure NC-induced ROS release because of the capacity of these cells to produce and modulate ROS in a dose-dependent manner [28,29]. The NCs were incubated with the cells at three different concentrations for short (1 and 3 h) and long (6 and 14 h) periods. Due to the higher toxicity of INU/pArg NCs, the concentrations tested for this prototype were 100, 50, and 10 µg/mL, while for CS NCs, concentrations were 200, 100, and 50 µg/mL. After a short incubation time (Supplementary Figure S3), the NCs were not able to induce a significant production of ROS at the concentrations tested. However, after incubation for 6 h, CS NCs induced low ROS levels at a concentration of 200 µg/mL (Figure 5). After 14-h incubation time, CS induced ROS release at concentrations of 50 µg/mL and 200 µg/mL, and INU/pArg NCs at 100 µg/mL, respectively (Figure 5). 

Moreover, both types of NCs also induced ROS in the A549 pulmonary cell line at the highest concentration tested after short incubation times (1 h and 3 h, Supplementary Figure S4), which could be related with the higher toxicity of the NCs on these lung epithelial cells, in comparison with the macrophage cell lines.

### 3.4. Complement Activation in Human Plasma Induced by the Polymeric Nanocapsules

**Figure 6:**
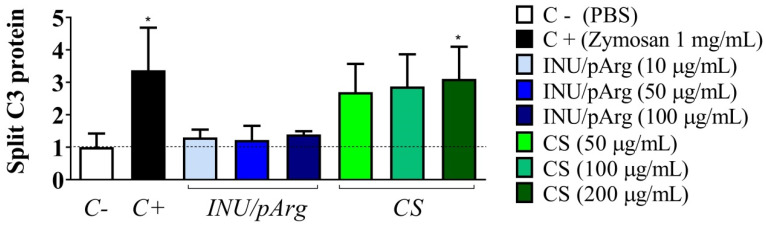
Relative quantification of the C3 complement factor protein degradation induced by the Imiquimod-loaded CS and INU/pArg nanocapsules at three different concentrations. CS: Chitosan, INU: Inulin, pArg: Polyarginine. Statistical significance: * P ≤ 0.05.

The main role of the complement system is to eliminate pathogens, by opening pores on their surfaces, but also to participate in the pathogen opsonization followed by phagocytosis and induce a series of inflammatory mediators that help to fight infection [30]. These mediators enhance both humoral (Th2) and cellular (Th1) immunity. For this reason, the activation of the complement system is a crucial step on the innate and acquired immunity to fight against pathogens. In fact, in vitro activation of this system, induced by nanostructure-based vaccines, has been correlated with a good immune response in vivo [31,32]. Hence, degradation of the C3 factor, a common step in all the complement activation pathways (mannose-binding-lectin, classical, and alternative pathways), was determined by Western blot in a pool of plasma incubated with the NCs (Figure 6) [30]. Only CS NCs induced a dose-dependent C3 protein degradation, which was statistically significant at 200 µg/mL and similar to the one induced by the positive control (Zymosan at 1 mg/mL). Hence, CS NCs induce activation of the complement system, possibly through the MB-lectin or alternative pathways, due to their polysaccharide composition. 

### 3.5. Cytokine Release Induced by the Polymeric Nanocapsules

Some cytokines are able to polarize the activated T helper (Th) lymphocytes into a Th1 (cellular) or Th2 (humoral) phenotype, or the macrophages into a M1 (pro-inflammatory) or M2 phenotype (anti-inflammatory) [33].

The analysis of the cytokine profile induced after vaccination or infection is complex and, therefore, it is difficult to predict the type of immune response that will be generated based only in this profile. Moreover, the development of Th1 and Th2 effector lymphocytes will also depend on the antigenic stimulation [34]. However, there is a clear evidence that IFN-γ and/or IL-12 polarize Th cells towards a Th1 profile, while IL-4 induces a Th2 phenotype [35]. Hence, a panel of cytokines involved in Th1 (IL-2, IL-12p70, INF-γ, and TNF-α) and Th2 immune response (IL-4, IL-5, IL-6, and IL-10) were tested. In addition, cytokines involved in inflammation, Th17 immune response, and cell proliferation, such as IL-1β, IL-17, and GM-CSF, respectively, were also studied [36]. 

**Figure 7:**
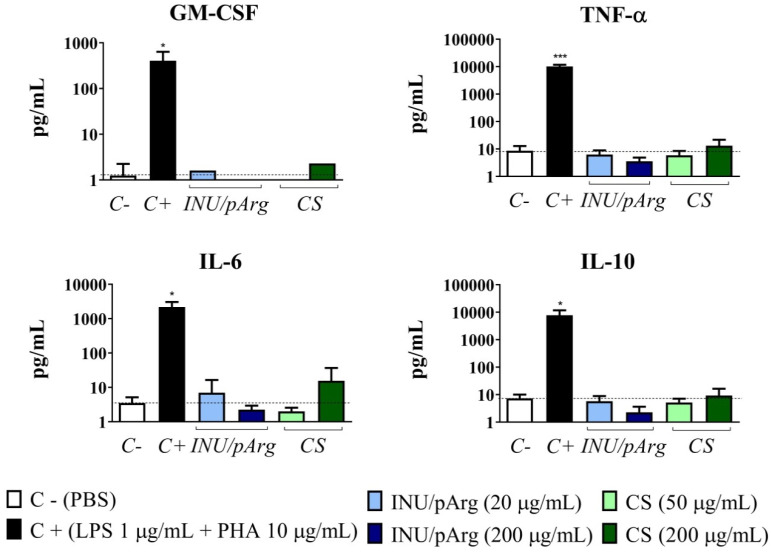
GM-CSF, TNF-α, IL-6, and IL-10 concentrations induced by Imiquimod-loaded CS and INU/pArg nanocapsules in PBMCs from healthy donors after 24 h of incubation. PBS was used as negative control and LPS+PHA as positive control. GM-CSF: granulocyte monocyte-colony stimulating factor, TNF-α: tumor necrosis factor alpha, IL-6: interleukin 6, IL-10: interleukin 10, CS: Chitosan, INU: Inulin, pArg: Polyarginine. Statistical significance: * P ≤ 0.05, *** P ≤ 0.001.

The polymeric NCs induced low levels of TNF-α, GM-CSF, IL-6, and IL-10 (Figure 7) and undetectable or very low levels of all the other cytokines tested. CS NCs at 200 µg/mL produced the highest concentrations but the difference with the negative control was not statistically significant for any of them. 

### 3.6. Systemic Immune Response against the ESAT-6/CFP-10 Fusion Protein after Intranasal Immunization with the Polymeric Nanocapsules

#### 3.6.1. Characterization of Specific IgG and IgA Antibodies against ESAT-6/CFP-10

A preliminary experiment was carried out to test the immunogenicity of the NCs, alone or in combination with the BCG vaccine. We selected a model vaccine against the ESAT-6 and CFP-10 antigens of Mtb, two early secreted antigens that are not present in the BCG vaccine, synthetized as a fusion protein (ECH protein). Hence, immunization with this fusion protein, in combination with the BCG vaccine, could induce specific humoral and cellular immune responses against Mtb to complement the limited protection induced by the licensed vaccine.

**Figure 8:**
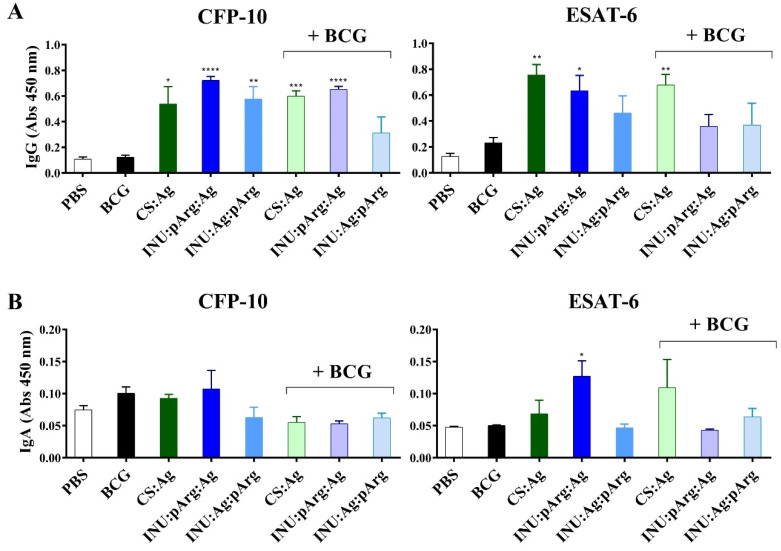
Serum IgG (**A**) and IgA (**B**) titers against CFP-10 and ESAT-6 measured in sera and BAL diluted 1/10 and 1/3, respectively, after three intranasal immunizations with the CS:Ag, INU:pArg:Ag, and INU:Ag:pArg nanocapsules in naïve mice or in mice previously immunized subcutaneously with the BCG vaccine (+BCG). Mice treated with PBS (PBS) or only immunized with BCG (BCG) were used as control groups. Ag: Antigen, BAL: bronchoalveolar lavage fluid, CS: Chitosan, Immunoglobulin (Ig), INU: Inulin, pArg: Polyarginine. Statistical significance: * P ≤ 0.05, ** P ≤ 0.01, *** P ≤ 0.001, **** P ≤ 0.0001.

It is worthwhile to mention that no relevant signs of toxicity, such as weight lost, impaired breathing, or death, were detected in the animals vaccinated with any of the NC vaccine prototypes. Moreover, no macroscopic changes were observed in any of the organs analyzed after the culling. The humoral response against the individual antigens (ESAT-6 and CFP-10) was characterized in the sera (IgG) and in the BAL (IgA) of animals immunized with the polymeric NCs by the i.n. route and compared to the control group (PBS) and the group immunized subcutaneously with the BCG vaccine (Figure 8). Specific IgG and IgA titers were determined by ELISA. The three NC prototypes induced significant IgG titers against both antigens (except for INU:Ag:pArg against the ESAT-6 protein) compared to the BCG and the PBS group. 

Regarding IgA titers, in general, they were insignificant and only the INU:pArg:Ag prototype induced specific IgA antibodies against the ESAT-6 antigen. Interestingly, for the CS:Ag NC prototype, previous priming with the BCG vaccine induced similar or even higher antibody titers, but the contrary was observed for both INU/pArg NC prototypes. In general, previous immunization with the BCG vaccine reduced the antigenicity of the INU:pArg:Ag and INU:Ag:pArg prototypes (Figure 8). 

Moreover, the prototype with the antigen on the surface (INU:pArg:Ag) induced higher antibody levels against ESAT-6, both for IgG and IgA, than the prototype with the antigen protected by the polymeric shell (INU:Ag:pArg). 

#### 3.6.2. Characterization of the Cellular Immune Response in Splenocytes from Immunized Animals

Apart from the humoral response, the nanovaccine prototypes were also tested for their capacity to induce a specific cellular immune response (Th1 or Th17). In fact, the IMQ adjuvant has been described to induce Th1 polarization through stimulation of the TLR-7 receptor [37]. Accordingly, an antigen recall experiment was carried out with splenocytes from immunized animals to measure IFN-γ, TNF-α, and IL-17 production, which are secreted from Th1 and Th17 subsets. The splenocytes were incubated with CFP-10, ESAT-6, or the ECH fusion protein, and the supernatants were collected for cytokine measurement. 

**Figure 9:**
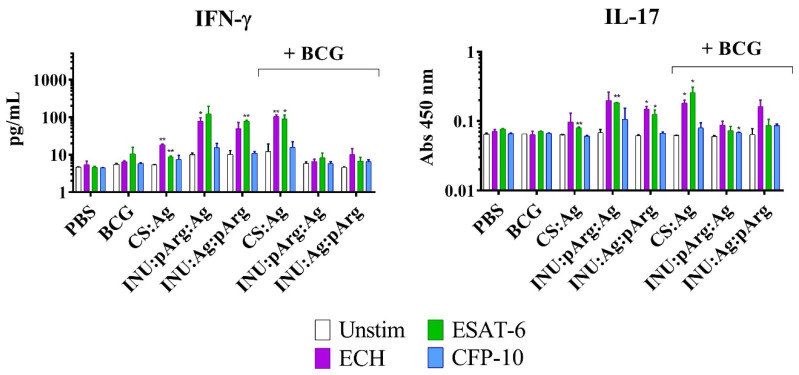
IL-17 and IFN-γ release from splenocytes of immunized mice after an antigen in vitro recall experiment. The splenocytes were incubated with CFP-10, ESAT-6, or the ESAT-6/CFP-10 fusion protein (ECH) and the supernatants were collected for cytokine measurement and were compared to the non-stimulated (Unstim) samples. Mice previously immunized with BCG (+BCG) or not, received PBS (as negative control), CS:Ag, INU:pArg:Ag, or INU:Ag:pArg as vaccine prototypes. IL-17: interleukin 17, IFN-γ: interferon gamma, Ag: Antigen, CS: Chitosan, INU: Inulin, pArg: Polyarginine. Statistical significance: * P ≤ 0.05, ** P ≤ 0.01, *** P ≤ 0.001.

Interestingly, only the ESAT-6 and the ECH fusion protein were able to induce IFN-γ and IL-17 release in animals immunized with the CS and the INU/pArg prototypes (Figure 9). The antigen recall induced the highest IFN-γ concentration in naïve animals immunized with the INU/pArg prototypes and in animals immunized with the CS:Ag NCs, but previously primed with the BCG vaccine. The same tendency was observed for IL-17, although the cytokine concentration was very low in all the groups. In contrast, TNF-α was not detected in any of the groups tested (data not shown). Hence, the polymeric NC vaccines were able to induce IFN-γ and IL-17 release, a Th1 and a Th17 activator of cytokines, respectively, but only against the ESAT-6 antigen. The CFP-10 antigen induced only a weak production of IFN-γ in animals immunized with the INU:pArg:Ag NCs. 

## 4. Discussion

Three vaccine prototypes were designed and developed during this work, using CS or INU/pArg NCs as antigen nanocarriers. IMQ was incorporated in the oily core as an additional adjuvant. The IMQ-loaded CS:Ag, INU:pArg:Ag, and INU:Ag:pArg NCs showed a similar particle size of about 150 nm, appropriate for nasal administration. In this sense, for an efficient delivery through the mucosal routes, it was stablished that the particle size should be smaller than the mucus mesh size [38]. This easily explains why NPs, in general, performed better than microparticles [39,40,41,42,43]. However, in the nanometric range, in vivo data has shown that very small nanometric size (30 nm) may be detrimental compared to larger ones (200 nm) [44]. Besides, not only the particle size, but also the composition, play a critical role when using NPs for nasal administration. In 1998, Alonso’s group reported the importance of PEG to increase the mucopermeation of polyester-based NPs [45], and, after that, many other studies confirmed the importance of an adequate PEG coating to favor the diffusion across the mucus layer [46,47].

On the other hand, the nanometric size of the developed NCs was also adequate to have a preferential internalization by dendritic cells (DCs) [6,48]. DCs induce a better T-cell stimulation than other APCs, such as macrophages and B-cells, and play a relevant role in pathogen capture and induction of a primary immune response. For that reason, particle vaccines try to target these cells [49]. Moreover, the NCs had a low (CS:Ag) or high (INU:pArg:Ag and INU:Ag:pArg) positive charge, which is also useful for the interaction with the negatively charged mucin following internalization in the mucosa [47,50]. 

The selected antigen was a fusion protein (ECH) of two well-known Mtb antigens, ESAT-6 and CFP-10. Both proteins belong to the region of difference (RD1) of Mtb, which is absent from all strains of BCG and is related with the virulence of the Mtb and *M. bovis* strains. In fact, the ESAT-6/CFP-10 protein complex could be related with macrophage apoptosis during TB infection [22]. The ECH recombinant protein is a potent immune activator for both humoral and cellular immune responses. Moreover, the i.n. administration of the recombinant protein in mice, in combination with and adjuvant, promoted the cellular immune response in comparison with the s.c. or intramuscular (i.m.) immunization routes that induced higher antibody and lower cellular immune responses [51]. Besides, it has been recently described that innate immune cells, monocytes/macrophages, and natural killer (NK) cells can also be trained through vaccination, pathogen colonization, or even previous infections [52]. These trained cells display stronger activation after a second exposure to the microbial molecules. Hence, activation of a broad set of immune cells could be the key to fight those pathogens that have a complex infection cycle.

Based on these results, the objective of this work was to investigate the potential of two different polymeric NC, made of two cationic polymers, CS and pArg, as carriers for the i.n. delivery of the ECH fusion protein, and if a previous boost with the BCG vaccine could improve the immune response by training the innate immunity. To increase the adjuvancy of the NCs, IMQ, a TLR-7 binding agonist, was encapsulated in the oily core of the NCs. The binding of this agonist to TLR-7 in APCs induces the release of pro-inflammatory cytokines, such as IFN-α, TNF-α, and IL-12, which, in turn, promote differentiation of immature Th cells into Th1 effector cells [37].

The release of TNF-α, IL-6, and IL-10 has been previously described for IMQ-loaded CS NCs, but not for empty CS NCs [10], and it can be associated to the internalization of the NCs and the stimulation of the TLR-7 receptor induced by the IMQ, as previously described for this immunostimulant molecule [37]. The differences in cytokine concentration found between the CS and the INU/pArg NCs could be due to differences in the internalization of the NCs or in the release of the IMQ. Low cytokine levels induced by IMQ-loaded CS NCs in vitro were also described in a previous work [11].

The stability of the formulations at storage conditions (4 °C) was good for all the prototypes, or the NCs before the addition of the antigen, in the case of CS NCs, but further lyophilization studies should be carried out for a final prototype. In fact, the lyophilization of the INU/pArg prototypes using 10% sucrose, as cryoprotectant, showed an efficient particle recovery after resuspension of the lyophilized formulation in water. 

CS and INU/pArg NCs showed a good cytocompatibility, although the toxicity was lower for CS vs. INU/pArg NCs. These results were in line with previous findings regarding the higher toxicity of pArg NCs than CS NCs [5,11]. On the other hand, INU NCs, without the pArg layer, showed considerably lower toxicities [53,54]. In addition, the higher IMQ content in the INU/pArg NCs could also be related with the increased toxicity [10].

Besides a higher cytocompatibility, CS NCs showed also a superior immunogenicity in vitro based on the induction of cytokine and ROS release and complement activation, all of them mechanisms that are related with an improved stimulation of the innate and adaptive immunity and associated efficacy in vivo [5,26,31,32]. 

However, from the in vivo experiments, it can be concluded that INU:pArg:Ag NCs were the only prototype able to induce specific IgA antibodies against the ESAT-6 antigen in mice immunized three times intranasally with the NCs. Moreover, the INU:pArg:Ag and the CS:Ag NCs induced high levels of IgG antibodies against the ESAT-6 and CFP-10 proteins, and IFN-γ and IL-17 release in splenocytes re-stimulated with ESAT-6 or the fusion protein. In contrast, protection of the antigen in a bilayer disposition (INU:Ag:pArg), despite increasing the stability of the NCs and the antigen loading compared to the INU:pArg:Ag NCs, reduced significantly the immune response. The placement of the antigen on the surface increased probably B-cell activation, as described previously [55,56]. Yet, the polymeric composition played also a significant role because the CS:Ag NCs failed to induce specific IgA antibodies although the antigen loading was also higher than for the INU:pArg:Ag.

Interestingly, the priming of the animals with the BCG vaccine (s.c. injected) only increased or maintained the immune response induced by the CS:Ag NCs, while, in general, it decreased or interfered with the response induced by the INU/pArg prototypes. In agreement with previous studies, combining different routes of immunization can interfere on the mucosal immune response [57]. In general, priming at the mucosal level and boosting through a systemic route has shown to induce a higher mucosal protection. However, this should be tested on individual prototypes because, in the case of the CS NCs, the priming with BCG did not interfere in the immune response induced by the NCs alone, and even increased the cytokine release in re-stimulated splenocytes from vaccinated animals.

The studies of the immune response against the ECH fusion protein and the individual components showed that ESAT-6 was a better antigen than CFP-10. The ESAT-6 protein was able to induce some specific mucosal IgA production in animals vaccinated with the INU:pArg:Ag NCs, and IL-17 and INF-γ release in splenocytes from animals immunized with the three vaccine prototypes after antigen recall, while the CFP-10 protein induced minor or undetectable levels. Although Th17 and Th1 linages are differently activated by ESAT-6, it has been shown in mice and human a developmental plasticity of the Th17 cells that enable a shift from dominant IL-17 secretion to dominant IFN-γ expression cells [58,59]. Hence, a combined immune response involving Th2 and either independent Th1 and Th17 and/or Th1-Th17 cells was elicited by the polymeric NCs against the ESAT-6 antigen, based on antibodies’ detection in animal serum and BAL, and cytokine release (IFN-γ and IL-17) in splenocytes after the antigen recall experiment. In contrast, a clear Th2-type immune response was observed for the CFP-10 antigen, based on antibody production.

The mucosal protection against ESAT-6, an Mtb-specific antigen that is not present in the BCG vaccine, could help in the neutralization of the pathogen at the route of entrance. However, to achieve a good immune protection for most of the population other antigens are needed. A recent computational study on the immunogenicity potential of 10 antigens from Mtb predicted that Rv2608 could be the most efficient one, based on the promiscuity of the epitopes to bind diverse human leukocyte antigen (HLA) molecules that are frequent in the population of three high-TB-burden countries [60].

Besides, to avoid the inhibition of the immune response induced by the INU:pArg:Ag NCs in animals previously immunized with the BCG vaccine, other vaccination regimes should be tested, such as the priming through the i.n. route with the NCs and the boosting through the s.c. route with the BCG vaccine.

## 5. Conclusions

Two different IMQ-loaded NCs, with a CS or an INU/pArg polymeric shell, were synthesized for the development of an i.n. model vaccine containing a recombinant fusion protein (ECH) derived from the ESAT-6 and CFP-10 antigen of Mtb. The vaccine containing INU:pArg:Ag NCs was the most immunogenic prototype against the ECH fusion protein. Further optimization of the NCs to increase the cellular response might provide an efficient antigen carrier for the development of new i.n. vaccines to fight pathogens affecting the mucosa and airways. 

Due to the interference of the BCG priming on the immune response, the use of INU:pArg:Ag NCs for priming and, afterwards, a boost with the INU:pArg:Ag NCs or the conventional BCG vaccine (s.c.) could improve the results observed for this prototype. In addition, antigen optimization should also be considered to obtain a more efficient vaccine against Mtb because CFP-10 induced lower cellular and humoral immune responses than the ESAT-6 protein.

## Figures and Tables

**Table 1:** Chemical composition of the CS and INU/pArg nanocapsules (NCs) developed in this work. CS: Chitosan, INU: Inulin, pArg: Polyarginine.

Chemical Composition	CS NCs	INU/pArg NCs
Oil	Miglyol/linoleic acid	Miglyol/linoleic acid/glycerin
Adjuvant	Imiquimod	
Polymer	Chitosan	Modified inulin
Surfactant	18:0 PE-PEG1000	
Co-surfactant	Sodium cholate	Sodium glycocholate
Second polymeric shell	-	Polyarginine
Antigen disposition	Surface	Surface/Polymer bilayer

**Table 2:** Physicochemical characterization, Imiquimod encapsulation efficiency, and antigen absorption of the different nanocapsules’ prototypes.

Nanocapsules	Size (nm)	Z (mV)	IMQ %EE	%AA
CS	145 ± 14	+28 ± 4.5	60 ± 4	n.a.
CS:Ag	152 ± 15	+8 ± 10	n.a.	60 ± 6
INU	158 ± 7	−37 ± 4	69 ± 6	n.a.
INU:Ag	136 ± 7	−36 ± 6	-	n.d.
INU/pArg	158 ± 22	+20 ± 1	-	n.a.
INU:Ag:pArg	151 ± 11	+27 ± 8	-	55 ± 5
INU:pArg:Ag	158 ± 12	+31 ± 7	-	30 ± 10

IMQ EE: Imiquimod encapsulation efficiency, AA: Antigen absorbed on the NCs. Polydispersity index (PdI) ≤ 0.2 in all the size measurements; n.a.: Not applicable; n.d.: Not determined. Ag: Antigen, CS: Chitosan, INU: Inulin, pArg: Polyarginine.

**Table 3:** **Half-maximum inhibitory concentration** (IC50) values of IMQ-loaded CS and INU/pArg nanocapsules in four different cell lines incubated with the nanocapsules for 24 h. The corresponding IMQ concentration is also indicated. CS: Chitosan, IMQ: Imiquimod, INU: Inulin, pArg: Polyarginine.

Cell Line	IC50 (µg/mL)			
	CS		INU/pArg	
	NCs	IMQ	NCs	IMQ
RAW 264.7	455	2.96	117	2.05
THP-1	810	5.26	192	3.36
NCI H460	226	1.47	57	1.00
A549	197	1.28	31	0.54

## Supplementary Materials

The following are available online at https://www.mdpi.com/1999-4923/12/6/489/s1. Figure S1. Size (bars), PdI (lines and symbols) (A), and Z-potential (B) of the chitosan (CS) nanocapsules with different ratios of antigen (Ag) adsorbed on the surface at three different time points (0, 4, and 7 days). Size and PdI were measured in ultrapure water and Z-potential in 1 mM KCl. Figure S2. Size (bars), PdI (lines and symbols) (A), and Z-potential (B) of the CS:Ag, INU:Ag:pArg and INU:pArg:Ag nanocapsules before and after lyophilization with 10% sucrose as cryoprotectant. Size and PdI were measured in ultrapure water and Z-potential in 1 mM KCl. Ag: Antigen, CS: Chitosan, INU: Inulin, pArg: Polyarginine. Figure S3. Absence of ROS release in HL-60 cells incubated with the CS or the INU/pArg nanocapsules at four different concentrations for 1 h (left panel) and 3 h (right panel). Cells incubated with RPMI medium alone (C−) or with zymosan (C+) were used as negative and positive controls, respectively. Figure S4. ROS release in A549 cells incubated with the CS or the INU/pArg nanocapsules at two different concentrations for 1 h (left panel) and 3 h (right panel). Cells incubated with RPMI medium alone (C−) or with zymosan (C+) were used as negative and positive controls, respectively.
