# Supplementary material for: Design of Polymeric Nanocapsules for Intranasal Vaccination against Mycobacterium Tuberculosis: Influence of the Polymeric Shell and Antigen Positioning
Source: Pharmaceutics. 2020 May 28;12(6):489. doi: 10.3390/pharmaceutics12060489 (PMC7355676; doi:10.3390/pharmaceutics12060489)
Supplement: Supplementary file 1 [file pharmaceutics-12-00489-s001.pdf]

pharmaceutics

MDPI

Supplementary Materials: Design of Polymeric Nanocapsules for Intranasal Vaccination against Mycobacterium Tuberculosis: Influence of the Polymeric Shell and Antigen Positioning

Lara Diego-González, José Crecente-Campo, Matthew John Paul, Mahavir Singh, Rajko Reljic, María José Alonso, África González-Fernández and Rosana Simón-Vázquez

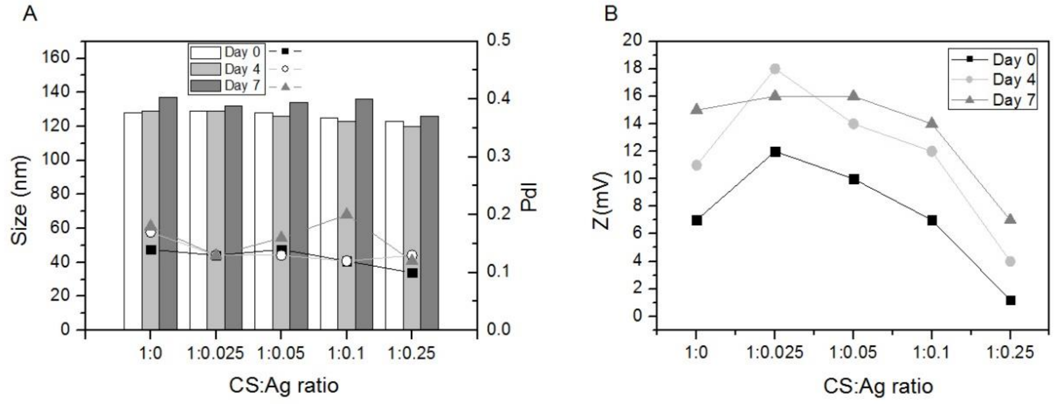

Figure S1. Size (Bars), polydispersity index (PdI) (lines and symbols) (**A**) and Z-potential (**B**) of the chitosan (CS) nanocapsules with different ratios of antigen (Ag) adsorbed on the surface at three different time points (0, 4 and 7 days). Size and PdI were measured in ultrapure water and Z-potential in 1 mM KCl.

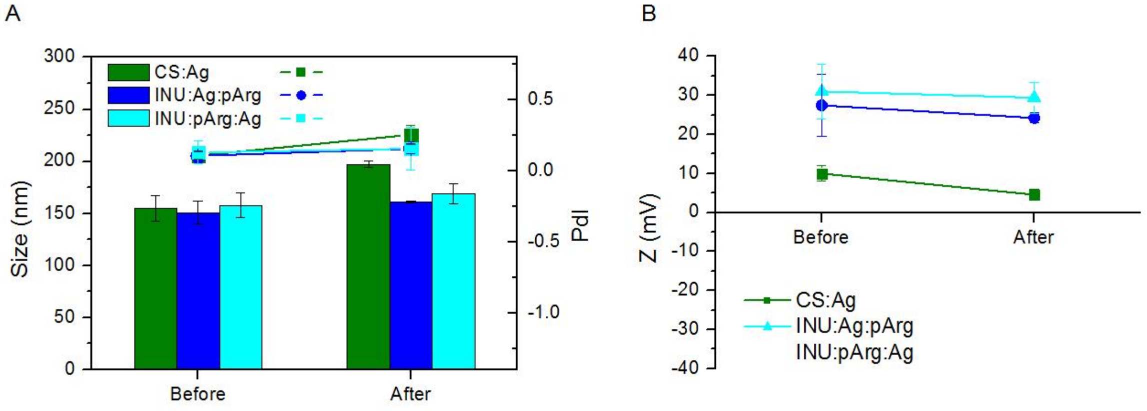

Figure S2. Size (Bars), PdI (lines and symbols) (**A**) and Z-potential (**B**) of the CS:Ag, INU:Ag:pArg and INU:pArg:Ag nanocapsules before and after lyophilisation with 10% sucrose as cryoprotectant. Size and PdI were measured in ultrapure water and Z-potential in 1 mM KCl. Ag: antigen, CS: chitosan, INU: inulin, pArg: polyarginine.

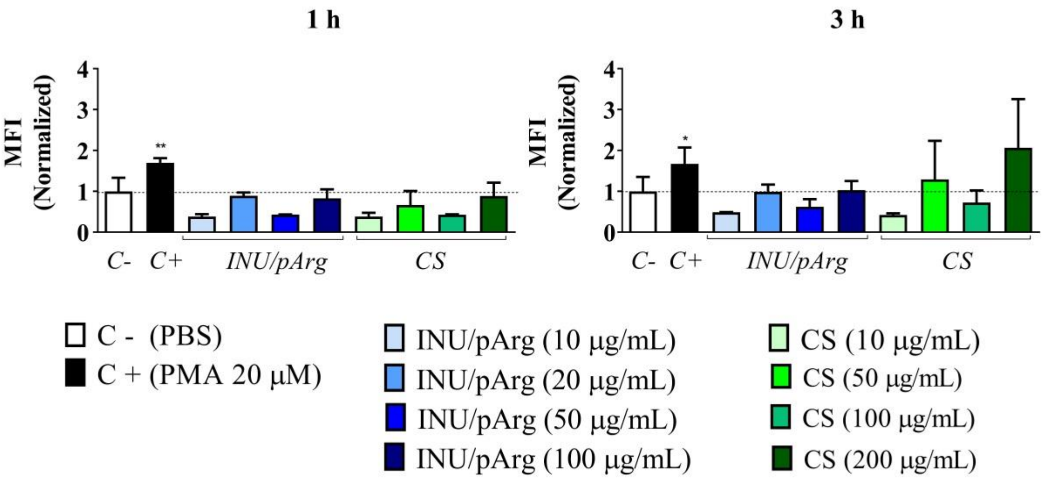

Figure S3. Absence of reactive oxygen species (ROS) release in HL-60 cells incubated with the CS or the INU/pArg nanocapsules at four different concentrations for 1 h (left panel) and 3 h (right panel). Cells incubated with RPMI medium alone (C−) or with zymosan (C+) were used as negative and positive controls, respectively. * P ≤ 0.05, ** P ≤ 0.01.

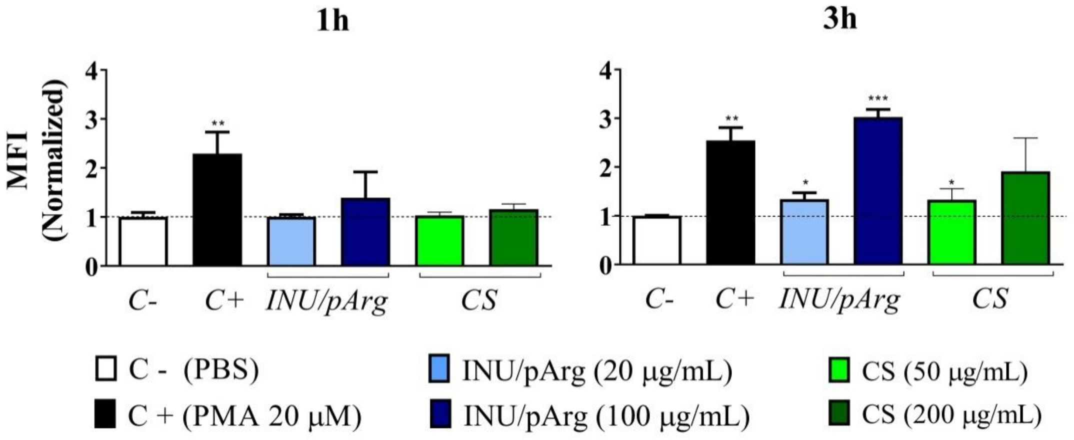

Figure S4. ROS release in A549 cells incubated with the CS or the INU/pArg nanocapsules at two different concentrations for 1 h (left panel) and 3 h (right panel). Cells incubated with RPMI medium alone (C−) or with zymosan (C+) were used as negative and positive controls, respectively. * P ≤ 0.05, ** P ≤ 0.01, *** P ≤ 0.001.
